# Genetic diversity and population structure assessment of Western Canadian barley cooperative trials

**DOI:** 10.3389/fpls.2022.1006719

**Published:** 2023-01-09

**Authors:** Ludovic J. A. Capo-chichi, Ammar Elakhdar, Takahiko Kubo, Joseph Nyachiro, Patricia Juskiw, Flavio Capettini, Jan J. Slaski, Guillermo Hernandez Ramirez, Aaron D. Beattie

**Affiliations:** ^1^ Department of Renewable Resources, Faculty of Agriculture, Life and Environmental Sciences, University of Alberta, Edmonton, AB, Canada; ^2^ Institute of Genetic Resources, Faculty of Agriculture, Kyushu University, Fukuoka, Japan; ^3^ Field Crops Research Institute, Agricultural Research Center, Giza, Egypt; ^4^ Field Crop Development Centre, Alberta Agriculture and Forestry, Lacombe, AB, Canada; ^5^ Ecosystems and Plant Sciences, InnoTech Alberta Inc., Vegreville, AB, Canada; ^6^ Department of Plant Sciences, College of Agriculture and Bioresources, University of Saskatchewan, Saskatoon, SK, Canada

**Keywords:** *Hordeum vulgare* L, historical datasets, SNP markers, allelic variability, association mapping, structure

## Abstract

Studying the population structure and genetic diversity of historical datasets is a proposed use for association analysis. This is particularly important when the dataset contains traits that are time-consuming or costly to measure. A set of 96 elite barley genotypes, developed from eight breeding programs of the Western Canadian Cooperative Trials were used in the current study. Genetic diversity, allelic variation, and linkage disequilibrium (LD) were investigated using 5063 high-quality SNP markers *via* the Illumina 9K Barley Infinium iSelect SNP assay. The distribution of SNPs markers across the barley genome ranged from 449 markers on chromosome 1H to 1111 markers on chromosome 5H. The average polymorphism information content (PIC) per locus was 0.275 and ranged from 0.094 to 0.375. Bayesian clustering in STRUCTURE and principal coordinate analysis revealed that the populations are differentiated primarily due to the different breeding program origins and ear-row type into five subpopulations. Analysis of molecular variance based on PhiPT values suggested that high values of genetic diversity were observed within populations and accounted for 90% of the total variance. Subpopulation 5 exhibited the most diversity with the highest values of the diversity indices, which represent the breeding program gene pool of AFC, AAFRD, AU, and BARI. With increasing genetic distance, the LD values, expressed as r^2^, declined to below the critical r^2 =^ 0.18 after 3.91 cM, and the same pattern was observed on each chromosome. Our results identified an important pattern of genetic diversity among the Canadian barley panel that was proposed to be representative of target breeding programs and may have important implications for association mapping in the future. This highlight, that efforts to identify novel variability underlying this diversity may present practical breeding opportunities to develop new barley genotypes.

## Introduction

Cultivated barley is among the oldest domesticated plants (Von Bothmer and Komatsuda, 2010). It is originated from wild barley (*Hordeum vulgare L. ssp.*) and is distributed through the Middle East, mostly falling into different classes according to phenology, morphology, and end-use: two-row *vs*. six-row, winter types *vs*. spring types, hulled *vs.* hull-less, feed barley *vs.* malting barley (Blake et al., 2011). Barley is adapted to a wide range of environmental conditions: withstands dry-hot climates, extensive salinity and marginal soils, in addition to a broad range of soil pH conditions (Weltzien, 1988; Pasam et al., 2014; Elakhdar et al., 2022), and low temperatures which is mainly based on an adaptive response. The adaptive responses refer to hardening or cold acclimation that activates by growth at low temperatures (Stanca et al., 2003). Barley is grown through different agroecological zones, from 46° S in Chile to 70° N in Norway (Leff et al., 2004). The physiological, morphological, and functional adaptation in barley reveals the fundamentals of genetic diversity which may assist to elucidate the environmental adaptation of this plant (Graner et al., 2003). In 2022, Canada ranked 4^th^ for barley production globally after Russia, France, and Germany (Faostat, 2022). The total production of barley in Western Canada is estimated at 10,416,300 tonnes in the 2020 (Marta and Tricia, 2020). In the past, barley was used as human food but evolved mostly into a feed, and grain brewing (Baik and Ullrich, 2008; Graner et al., 2010). Due to the high levels of beneficial components for the human diet, barley now used as a functional food improvement (Dvořáková et al., 2008; Galli et al., 2020). The main advantage of barley in different food products and their consumption stems from barley’s potential health benefits. For example, several studies have shown that β-glucan soluble fiber from oat and barley can lower total and LDL cholesterol and thus play a role in both the prevention and management of cardiovascular disease (Ames and Rhymer, 2008).

Genetic improvement of crops depends on the level of variations in the germplasm which can be discovered by a specific and/or a combination of approaches (Mackay and Powell, 2007). Genetic diversity within a given gene pool reduces the vulnerability of crops to biotic and abiotic stress (Pasam et al., 2014). Hence, it is necessary to incorporate new sources of genetic diversity in breeding programs to achieve higher levels of tolerance to different environmental stress for increased yield. Studying genetic diversity is an essential tool for crop improvement through elucidating the diversity between the parental lines before the hybridization and introgression of desirable genes into elite genotypes (Chabane et al., 2005). The information provided by these studies has contributed to a better understanding of how germplasm collections are maintained over a long time (Mcclean et al., 2012). In association mapping studies, illustrating genetic diversity and population structure is also critical since the latter can lead to spurious associations (Gyawali et al., 2016). The benefit of this is particularly great when the dataset contains traits that time-consuming and/or are expensive to measure (Beattie et al., 2010). Thus, studying genetic diversity will support identifying genomic regions of related traits that control the phenotypic variation (Amezrou et al., 2017).

Several molecular markers have been used to measure genetic diversity, population structure, association studies, evolutionary origin, and breeding applications in barley. So far, simple sequence repeats (SSRs), Diversity arrays Technology (DArT), and single-nucleotide polymorphisms (SNPs) based on next-generation sequencing (NGS) have been utilized intensively in barley genetic diversity studies (Rafalski, 2002; Kumar et al., 2012; Elakhdar et al., 2016a; Elakhdar et al., 2016b; Elakhdar et al., 2018; Capo-Chichi et al., 2021). In barley, high-throughput SNP genotyping has revolutionized genome-wide association (GWAS) and gene mapping studies in recent years due to the development of high-throughput genotyping platforms. The barley 9 K iSelect Illumina SNP platform offers whole-genome coverage with enough genetic characterization, that will make the diversity contained in a given germplasm collection efficiently available to barley breeders (Malysheva-Otto et al., 2006; Comadran et al., 2009). Determining the density of markers necessary for association mapping or genomic selection is depending on linkage disequilibrium (LD) decay (Nordborg and Tavaré, 2002). Numerous studies of genetic diversity and LD have been highlighted in cultivated barley worldwide (Malysheva-Otto et al., 2006; Saisho and Purugganan, 2007) European genotypes (Rostoks et al., 2006), or North Africa (Elakhdar et al., 2016b; Elakhdar et al., 2018; Sallam et al., 2018). The results of these studies display a strong genetic structure within different collections of barley, with major groups corresponding to growth-habit and row-type (Elakhdar et al., 2018). Considering that barley is thought to have been domesticated twice, the differences in population genetic characteristics of these barley groups are likely to reflect their differences in breeding histories as well as differences in domestication histories (Morrell and Clegg, 2007).

To build on Canada’s position as a supplier of premium quality barley yield production, the Western Canadian cooperative trials breeding program aims to develop barley varieties with improved traits. For that purpose, a cooperative trial has been formalized in Western Canada in which 25 to 35 lines are evaluated annually in 15 to 20 locations. The result is an extensive collection of historical data demonstrating a wide variety of elite barley genotypes that can be used to discover desirable alleles through association mapping. With such unbalanced historical datasets, association mapping has been constructed for yield in barley (Kraakman et al., 2004; Beattie et al., 2010). The present study is the first to explore the genetic diversity within the Western Canadian barley cooperative trials collections using the Illumina iSelect SNP array. This study aims to (i) investigate the diversity patterns in 96 elite barley genotypes from eight breeding programs of the Western Canadian Cooperative Trials, (ii) assess LD decay in different subpopulations, and (iii) compare the level of polymorphism between the germplasm.

## Materials and methods

### Plant materials

A set of 96 elite barley genotypes from Western Canadian breeding programs were used in this study. These panel from eight barley breeding programs and were grown at different times between 1994 and 2016 (**Table 1**). The panel consisting of registered cultivars, advanced breeding lines, and two-row lines for study limit dextrinase and beta-glucanase that have been evaluated over 15 years in the Western Two-Row Cooperative registration (Beattie et al., 2010). The advanced promising lines were selected based on their high percentage seed yield. Ninety-two lines of this population were used in the previous project (FHB screening of CDC barley breeding selections, 2011-2016). The registered cultivars include well-known cultivars, ‘CDC Kendall’, ‘AC Metcalfe’, ‘Harrington’, ‘CDC Copeland’, ‘CDC Landis’, ‘CDC Meredith’, ‘Merit 16’, ‘CDC Reserve’, and ‘Bentley’.

**Table 1:** The breeding program origin for barley lines in the current study.

Breeding program origin	Number of lines	Cultivars
AAFRD Lacombe	13	
BARI	15	Merit 16, Merit, Merit 57, Conrad
Coors	4	Moravian 19, Moravian 27, Moravian 22, Moravian 28
AAFC Lethbridge	6	
CDC	28	CDC Aurora Nijo, CDC Bold, CDC Austenson, CDC Copeland, CDC Goodale, CDC Fraser, CDC Kendall, CDC Meredith, CDC Landis, CDC Reserve, CDC Mindon, CDC Unity, Harrington, CDC Select, Manley
AAFC Brandon	14	AAC Synergy, AC Metcalfe, Calder, AC Bountiful, Norman, Newdale
AU	7	Garnet
Cargill	4	
Landrace	5	

AAFRD, Alberta Agriculture, Food and Rural Development; BARI, Busch Agricultural Resources Inc; AAFC, Agriculture and Agri-Food Canada; CDC, Crop Development Centre, University of Saskatchewan; AU Agricore United; BARI, Busch Agricultural Resources Inc.

### Genomic and SNP genotyping

Genomic DNA was collected from the young leaf tissue from each of the 96 genotypes using the DNeasy Plant Mini Kit (Qiagen, Hilden, Germany), then quantified at 230/260 and 260/280 absorption ratios. The barley panel was genotyped using an Illumina 9K barley Infinium iSelect SNP assay at the USDA-ARS Genotyping Laboratory, Fargo, ND, USA. The 9K barley SNP assay can interrogate approximately 9,000 SNP markers, containing the 1,596 SNPs on BOPA1 loci (https://bioinf.hutton.ac.uk/iselect/app/index.pl). The markers with unknowing chromosomal positions, monomorphic or had a minor allele frequency of<0.05, and SNPs with missing values of more than 20% of the genotypes were removed. Only, the markers with genetic and physical position information on the 9K SNP array were used. A total of 5063 high-quality SNP markers remained in the dataset and was used for estimating the diversity analysis and population structure.

### Properties of SNP markers

Genetic diversity statistics for 5063 SNP markers including genetic diversity (GD), major allele frequency (MAF), heterozygosity, and availability were calculated (Weir, 1996) using PowerMarker v3.25 (Liu and Muse, 2005). The polymorphism information content (PIC) values for SNP data were estimated using the following formula (Botstein et al., 1980);


$$PIC=1-\sum_{i=1}^{n}p_{1}^{2}-\sum_{j=i+1}^{n}2P_{i}^{2}\;P_{j}^{2}$$

Gene diversity was calculated as, $H_{e}=1-\sum^{}p_{i}^{2}$ (Nei, 1978), where *pi* and pj are the frequency of the *ith* and *jth* alleles of a given locus, respectively.

### Genetic diversity and population structure analysis

The population structure analysis for the whole genotypes panel was estimated with 5063 SNP markers using STRUCTURE V2.3 (Pritchard et al., 2000). The Bayesian model-based clustering approach was used to define the number of subpopulations (*k*) given an admixture model with associated allele frequencies. STRUCTURE simulations were performed with the number of presumed clusters from *k*=1 to *k*=10. Five independent runs were used for each subpopulation (*k*-value). For each run, simulations were run for 100,000 burn-in and 100,000 Markov Chain Monte Carlo (MCMC) iterations (Tabanao et al., 2014). The Delta *k* statistic of (Evanno et al., 2005) was estimated using STRUCTURE HARVESTER (Earl and Vonholdt, 2011) to determine the best *k* value. The genetic relationships between groups were further analyzed by principal coordinate analysis (PCoA) and the percentage of variations using GenAlEx v6.5 (Peakall and Smouse, 2012).

### Analysis of molecular variance and genetic diversity indices

AMOVA was performed to separate molecular variance at two levels: (a) among subpopulations (genotypes) which were inferred by the analysis of population structure, and (b) within subpopulations. To further verify the genetic differentiation, the number of different (Na), and effective alleles (Ne), expected heterozygosity (He), Fixation index (F), Shannon’s Information Index (I), diversity among and within subpopulations, the unbiased diversity index (uh) and pairwise PhiPT values were calculated for each population according to (Smouse et al., 2015). The PhiPT value, an analog of F_st_, suppresses variance within populations while calculating differentiation between populations according to genotypic variance. To determine whether the variance component partitioning is significant, we estimated the probability values by 1000 permutations. The PhiPT values are calculated according to the formula; PhiPT = *AP*/(*WP* + *AP*), where AP; diversity among populations, WP; diversity within populations. In addition, the number of loci with private alleles, and the percentage of polymorphic loci were also computed using GenAlEx v6.5 (Peakall and Smouse, 2012).

### Linkage disequilibrium decay

Linkage disequilibrium (LD) was computed using the GAPIT program in R software (Clayton, 2015) as a pairwise squared allele frequency correlation (R^2^) for each chromosome. To associate the decay of LD among the genotypes, the genetic distance between each pair of SNP markers was plotted against R^2^ values. The squared allele frequency correlations (R^2^) for each pair of markers were measured to estimate significant linkage disequilibrium at a *p-value<*0.001. The density of the filtered SNP distribution on seven chromosomes was drawn using the CMplot package in R.

### Phenotypic correlation analysis

To demonstrate that the panel includes significant phenotypic variations for future use such as association mapping. Data have been collected on chlorophyll fluorescence-related traits; Minimum Fluorescence (F_0_), Variable fluorescence (F_V_), Maximum fluorescence intensity (F_M_), Maximum efficiency of Photosystem II (PSII), (F_V_/F_0_), and Maximum yield of primary photochemistry (F_V_/F_M_) under low-temperature stress (LTS). In brief, at a three-leaf stage the seedlings were exposed to temperatures raised between 3°C and 5°C, then gradually decreased to -12°C for 4 hours. Chlorophyll fluorescence parameters were measured as an indicator of photosynthetic energy that responds to alters in PSII photochemistry according to (Ghassemi-Golezani et al., 2008). Pearson correlation analysis between the chlorophyll fluorescence-related traits; F_0_, F_V_, F_M_, F_V_/F_0,_ and F_V_/F_M_ under low-temperature tolerance before and two hours after the treatment were calculated using ‘‘Hmisc’’ packages in R, the ‘‘PerformanceAnalytics’’ packages was used for drawing scatter plots.

## Results

### SNP markers quality distribution

**Figure 1:**
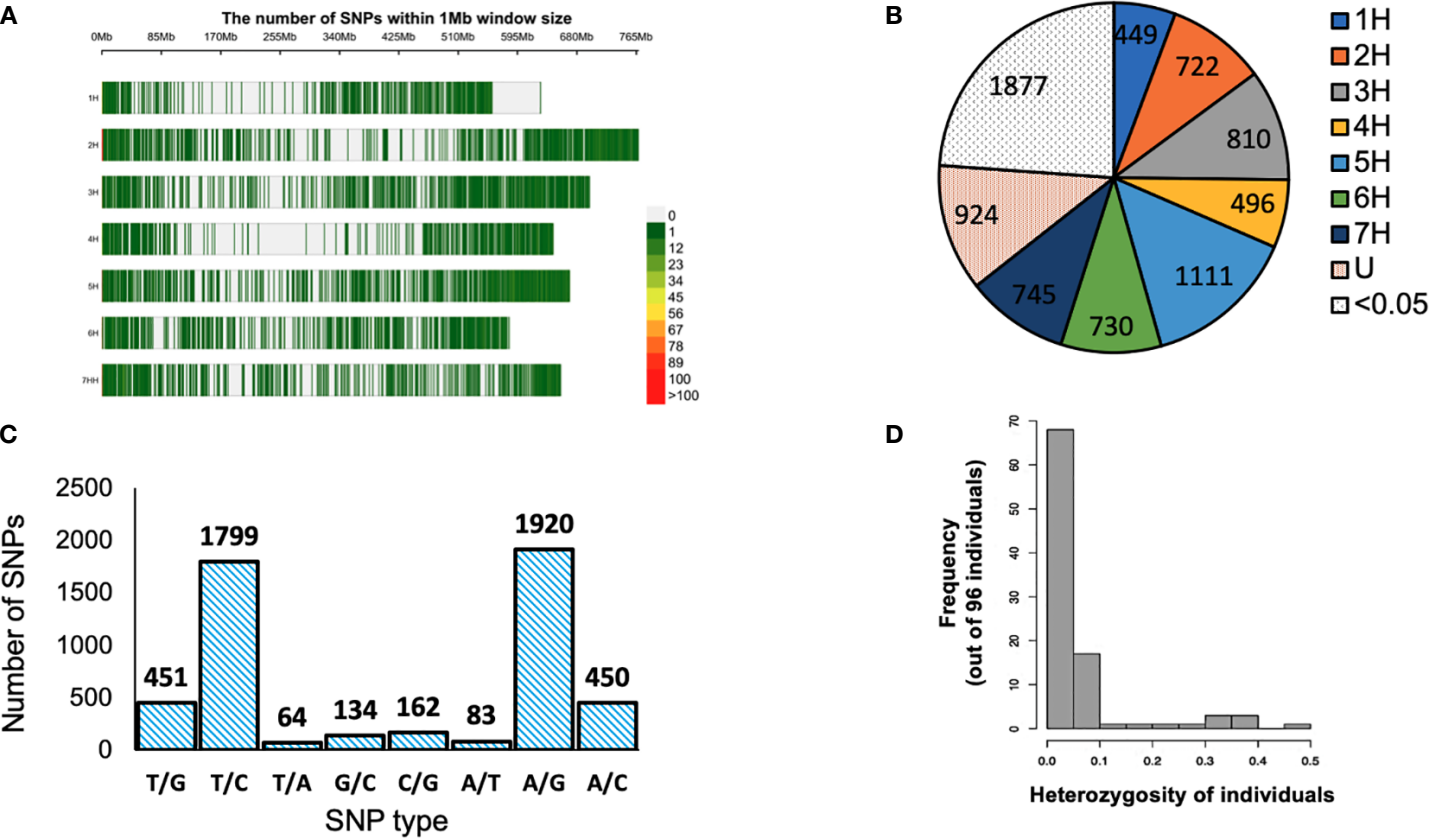
**(A)** Distribution of single nucleotide polymorphisms (SNPs) in the 9K Barley Infinium iSelect SNP assay. **(B)** Distribution of SNP markers obtained high quality filtration on the barley genome based on the physical map. U: markers with unknown positions SNPs with MAF of <0.05. **(B)** Distribution of SNP markers obtained high quality filtration on the barley genome based on the physical map. Color legend on the right shows the marker density. **(C)** SNP type for 5063 SNP markers used in the analysis. **(D)** Structure of heterogeneity detected in 96 barley genotypes > < 0.5. Color legend on the right shows the marker density. **(C)** SNP type for 5063 SNP markers used in the analysis. **(D)** Structure of heterogeneity detected in 96 barley genotypes > < 0.5. **(B)** Distribution of SNP markers obtained high quality filtration on the barley genome based on the physical map. Color legend on the right shows the marker density. **(C)** SNP type for 5063 SNP markers used in the analysis. **(D)** Structure of heterogeneity detected in 96 barley genotypes.

Ninety-six barley genotypes were used to elucidate the genetic diversity within the gene pool of the Western Canadian cooperative trials. This panel includes elite germplasms and key cultivars which have featured strongly in the development of the recent barley cultivars in Western Canada or the Canadian Prairies. The panel was genotyped by using a 9K Illumina iSelect SNP array that contained more than 7864 markers. After quality control filtering of the dataset, 5063 SNPs were used for analysis. For each SNP marker, chromosomal positions were obtained from the James Hutton Institute, Scotland (https://bioinf.hutton.ac.uk/iselect/app/index.pl). The high-quality SNPs were well distributed across the barley genome and presented in (**Figure 1A**). The highest number of markers was observed on chromosome 5H; 14.13% (1111 SNPs), while chromosome 1H had the lowest number of SNPs with a percentage of 5.71% (449 SNPs) among the seven chromosomes (**Figure 1B**). The frequency of A/G and T/C transitions had the highest frequency of 38% (1920) and 35% (1799), respectively. Though the lowest frequency among the eight alleles combination was T/A with 1% (64) (**Figure 1C**).

### Genetic diversity

The genetic diversity was assessed using the filtered 5063 SNPs markers. The GD values ranged from 0.099 to 0.500 with an average of 0.341 (**Table 2**). The average PIC was 0.275 and ranged from 0.094 to 0.375 in the barley genome (**Table 2**). The average MAF was 0.747 while the heterozygosity means value was 0.055. High genome-wide heterozygosity of SNP markers was observed for the 96 genotypes (**Figure 1D**).

**Table 2:** Estimation of major allele frequency, gene diversity, PIC and availability.

Marker quality and diversity	Minimum	Maximum	Mean
Major allele frequency	0.500	0.948	0.747
Gene Diversity	0.099	0.500	0.341
PIC	0.094	0.375	0.275
Availability	0.115	1.000	0.984

PIC, polymorphism information content.

### Population structure

**Figure 2:**
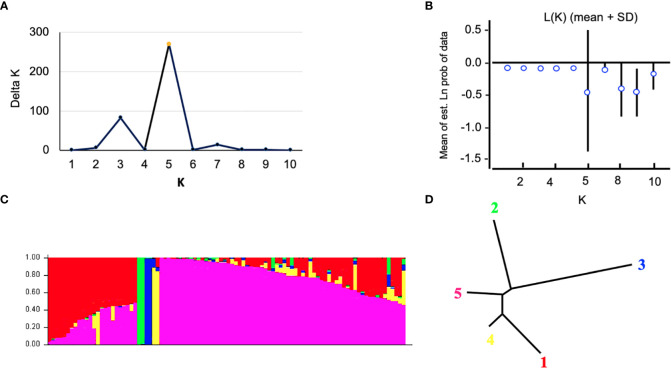
Analysis of population structure using 5063 SNP markers: **(A)** estimated population structure of 96 tow-rowed barley germplasm (k = 5). The y-axis the sub-population membership, and the x-axis is the genotypes, and **(C)** delta (Δ) K for different numbers of sub-populations, and **(B)** the average of the log-likelihood value. The mean values of ln P(D) and SD from 10 runs for each value of K 1/4 1–10. **(D)** Tree indicates the relationships among the five subpopulations.

The Bayesian model-based method was used to investigate the genetic structure of 96 Canadian barley genotypes using highly informative selected markers (5063 SNPs). To distinguish the optimum number of subpopulations, the number of clusters (*K*) was plotted against ΔK (**Figure 2A**). A gradual enhancement was detected in the calculated log-likelihood [Ln P(D)] by the growth in K (**Figure 2B**). The best number of K-values determined the number of populations as K = 5, depicting that those five subpopulations might contain all 96 genotypes with the highest probability (**Figures 2C, D**). Out of 96 genotypes, 24 genotypes were assigned to subpopulation 1 with 25% of the whole panel. While, subpopulations 2, 3, and 4 had two genotypes each with 2.08%, and 66 genotypes were assigned to subpopulation 5 with 68.75% (**Figures 2C, D**). Genotypes derived from the breeding programs of Coors, Cargill, and AU, and some of the AAFRD Lacombe genotypes exhibited highly diverse membership. Remarkable genetic divergence was observed among five subpopulations in addition to the average distance, He or gene diversity was detected among the germplasm in each subpopulation. The highest value of He was observed in subpopulation 5 with a value of 0.992 and the lowest was noted in subpopulation 3 with a value of 0.625. Furthermore, the highest value in diversity among and within subpopulations was observed in subpopulation 5 (**Table 3**).

**Table 3:** Structure analysis of 96 barley genotypes.

Subpopulations	No. of genotypes	He	F_st_	AP	WP
Pop1	24	0.973	0.028	3.135	0.759
Pop2	2	0.750	0.333	0.693	0.015
Pop3	2	0.625	0.600	0.693	0.015
Pop4	2	0.750	0.333	0.693	0.015
Pop5	66	0.992	0.008	4.190	2.911

He; expected heterozygosity, Fst; fixation index, AP; diversity among Pops, WP; diversity within Pops.

The results of the STRUCTURE estimated that the fixation index (F_st_) showed significant divergence within the subpopulations. A high level of genetic variation was observed in subpopulation 3 with the F_st_ value of 0.600. Subpopulation 5 displayed lower genetic variation with an F_st_ value of 0.008 among its genotypes (**Table 3**). Genotypes collected from the same breeding program commonly belonged to the same gene pools. Subpopulation 1 comprises the common individuals from the breeding program CDC. Nearly all remaining individuals from subpopulation 5 were assigned to the breeding program gene pool of AFC, AAFRD, AU, and BARI. Subpopulations 2, 3, and 4 originating from the AAFRD were very diverse, as their genotypes were attributed to dissimilar gene pools.

### Genetic differentiation and gene flow

The assessment of genetic variability was calculated within (intra) and among (inter) populations by an AMOVA. A two-level AMOVA of 96 barley genotypes, belonging to eight different breeding programs in Canada, revealed that 95% of the total genetic variation exists within populations and 5% among populations based on PhiPT values (**Table 4**). These results revealed that genetic differentiation within subpopulations was higher than among subpopulations. Pairwise PhiPT genetic distance values ranged from 0.001 between Pop1 and Pop5, and 0.444 between Pop1 and Pop3. PhiPT values were significant (*P*< 0.001) for all the subpopulation-pairwise comparisons (**Table 5**). Based on Nei’s minimum distance, a similar pattern of subpopulation differentiation was observed (**Table 5**). Pairwise genetic distances between populations were computed using GenAlEx v6.5, when *P-value*< 0·001. The genetic distances between populations collected from the different breeding programs were higher than 0.05. The maximum value of genetic distances between populations was 0.693 between Pop2 and Pop3; Pop2 and Pop4, while the lower value of 0.133 resulted between Pop3 and Pop5; Pop4 and Pop5 (**Supplemental Table 1**).

**Table 4:** Analysis of Molecular Variance (AMOVA) of western Canadian collections.

Source	df	SS	MS	Est. Var.	%	Stat		p value	p alpha
Among Pops	4	6286.195	1571.549	54.532	5%				
Within Pops	91	87803.867	964.878	964.878	95%	PhiPT	0.053	0.05	*p > 0.01*
Total	95	94090.063		1019.410	100%				

**Table 5:** Pairwise genetic distances between populations.

Subpopulations	Pop1	Pop2	Pop3	Pop4	Pop5
Pop1	0.000	0.118	0.444	0.005	0.001
Pop2	0.053	0.000	0.326	0.337	0.378
Pop3	0.000	0.061	0.000	0.350	0.436
Pop4	0.264	0.117	0.104	0.000	0.047
Pop5	0.057	0.010	0.000	0.098	0.000

PhiPT values (above diagonal) and Nei’s minimum genetic distance (below diagonal) between subpopulations. Probability based on 1000 permutations is shown above diagonal.

### Patterns of allele frequency across populations

**Figure 3:**
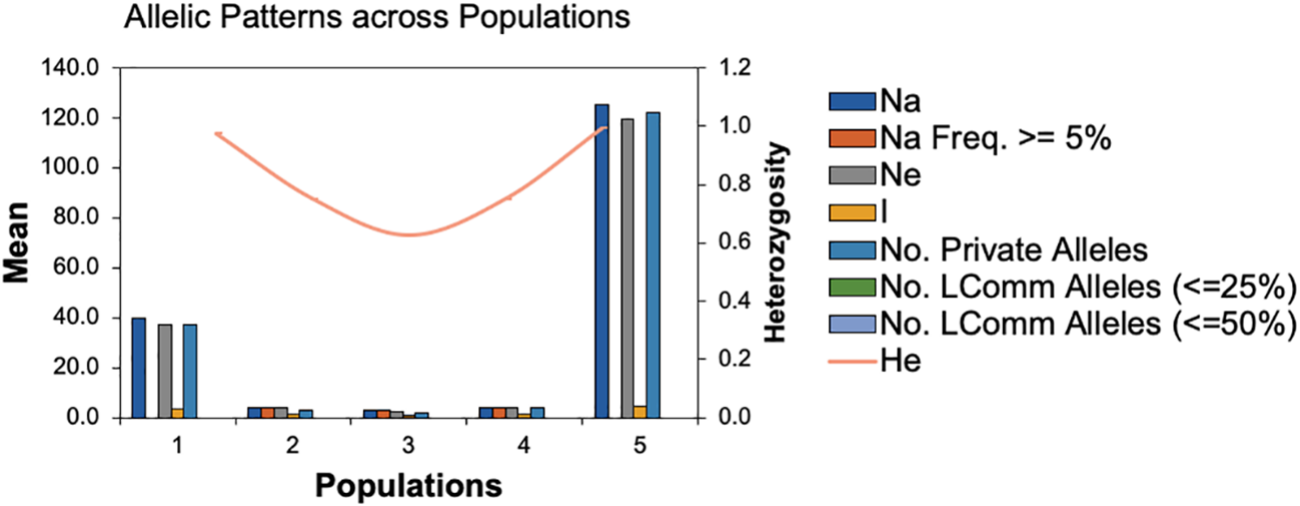
Patterns of allele frequency across populations. Na; no. of different alleles, Na (Freq >= 5%); No. of different alleles with a frequency >= 5%, Ne= No. of effective alleles, I; Shannon’s Information Index, No. LComm Alleles (<=25%); No. of Locally Common Alleles (Freq. >= 5%), No. LComm Alleles (<=50%); No. of Locally Common Alleles (Freq. >= 5%), He, Expected Heterozygosity; uHe, Unbiased Expected Heterozygosity.

To infer significantly different patterns and levels of population genetic structure, patterns of allele frequency across populations were calculated. The Na and Ne ranged from 30 to 125 and 2.667 to 119.342, respectively. While, the Shannon index (I) and the unbiased diversity index (uh) values ranged from 1.040 to 4.809 and 0.833 to 1.00, respectively (**Supplemental Table 2**). Furthermore, the statistical parameters of the mean and standard errors for each subpopulation across loci are summarized in **Figure 3**. All the results of allelic patterns revealed that subpopulation 5 is the most diverse population since it has higher diversity indices values and represents the breeding program gene pool of AFC, AAFRD, AU, and BARI. While, low diversity was observed in subpopulations; 3, 2, and 4, respectively as expected with their low number of genotypes.

### Principal coordinate analysis

**Figure 4:**
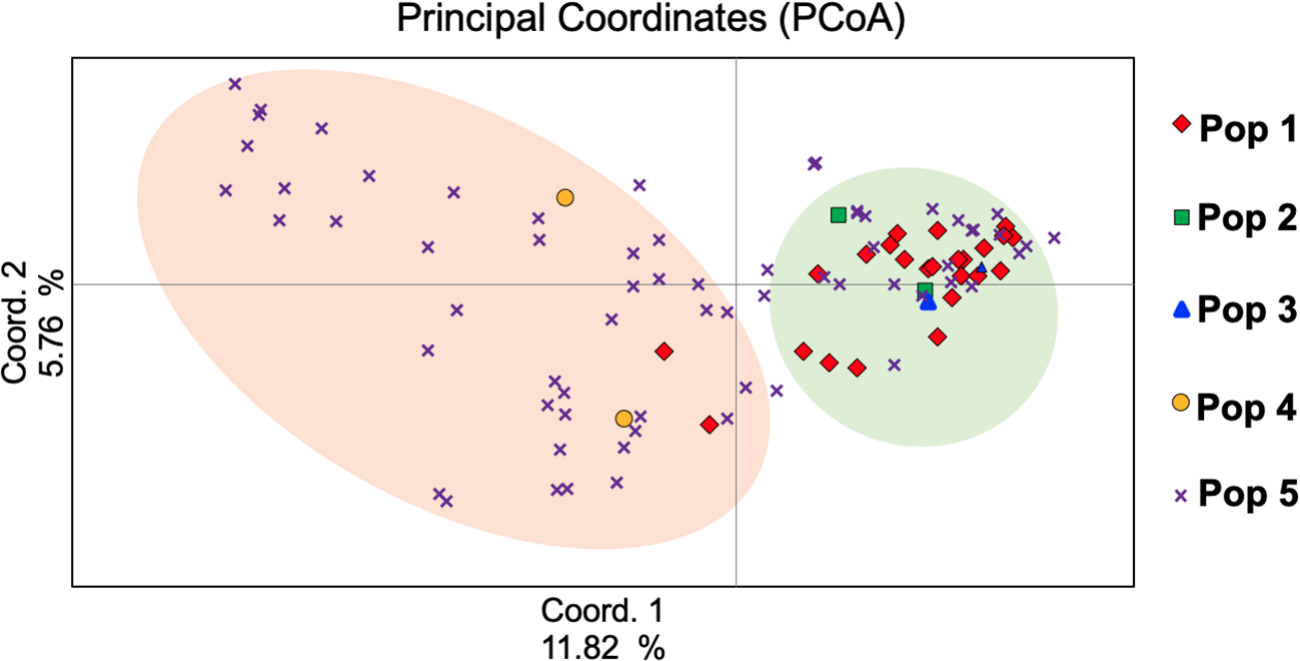
Principal coordinate analysis (PCoA) of the Western Canadian barley genotypes.

To understand the genetic relationships among the studied genotypes, PCoA was performed and the first three components of PCoAs explained 22.20% of the total genetic variation (**Figure 4**), with 11.82%, 5.76%, and 4.62%, respectively (**Supplemental Table 3**). The PCoA analysis matched population structure findings in that they revealed the relationships among genotypes. In subpopulation 5 of the program gene pool of AFC, AAFRD, AU, and BARI were highly distinct. Overall, 22.22% variation was detected by the first three PCoA (**Figure 4**), which indicates that genotypes from the eight-breeding program were diverse from one another.

### Analysis of linkage disequilibrium

**Figure 5:**
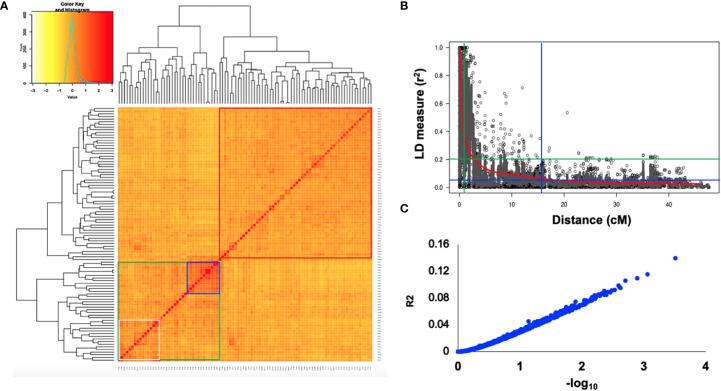
**(A)** Heat map of kinship matrix for the 5063 SNP markers. **(B)** Plots of LD values (r^2^) against genetic distance (cM) between pairs of SNPs. The red line indicates the non-linear regression line of the LD decay, the blue line displays the intersection of the 95% percentile representing the background LD, green line shows the intersection of an *r* ^2^ value of 0.2 with the regression line **(C)** R^2^ values against –log_10_ over all chromosomes.

Using the classical LD algorithms r^2^ (squared allele-frequency correlations) and D′ (the standardized disequilibrium coefficient), we investigated whole-genome patterns of LD using the mixed-model method and population structure as co-factors to understand the predictive value of a marker for the association between its chromosomal region with the phenotype. The r^2^ between each SNP pair (cM) was calculated to identify the level of co-occurrence of alleles at two loci. The SNP markers with *P< 0.001* of R^2^ on the seven barley chromosomes were selected for use in subsequent analysis. The mean values of R^2^ over all chromosomes revealed that there was no clear correlation with physical distance. A strong LD was observed among the 5063 marker pairs in the studied population with an approximate average value of 0.021 when the distance was 0.391 cM (**Table 6**). In contrast, the heatmap kinship of the distribution of intra-chromosomal r^2^ values across the whole genome of the tested genotypes highlights the extended LD values across the genetic centromeres (**Figure 5A**). Accordingly, the extent of LD was intensely influenced by population structure. A background of long-range LD groups was observed, that are usually due to the population structure and admixture within the genotypes (Ersoz et al., 2007). Furthermore, r^2^ methods with a maximum of 1 at two SNP pairs alleles display more frequent co-occurrence within the 96 genotypes (**Figure 5B**). Plotting LD of genetic distance showed comprehensive intra-chromosomal LD over each barley chromosome (**Table 6**). In all the barley genotypes analyzed, the maximum number of LD between two loci was on chromosome 5H with an average of 0.0141 and an R^2^ of 22.10%, followed by chromosome 3H. In contrast, the minimum number of LD was observed on chromosome 1H with an average of 0.0093 and an R^2^ of 8.79% (**Table 6**).

**Table 6:** Linkage disequilibrium between two polymorphisms alleles across the seven chromosomes.

Chromosome	1H	2H	3H	4H	5H	6H	7H
Number sig. LD	444	714	810	488	1116	725	744
Average Sig. LD	0.0093	0.0061	0.0126	0.0191	0.0141	0.0211	0.0114
Percentage R^2^	8.79	14.14	16.04	9.66	22.10	14.36	14.73
Whole panel							
Mean R^2^	0.021						
Mean D’	0.391						
Mean p-value	0.443						

D’; the standardized disequilibrium coefficient, R^2^; represents the correlation between alleles at two loci, which is informative for evaluating the resolution of association approaches.

### Correlations and phenotypic variation

**Figure 6:**
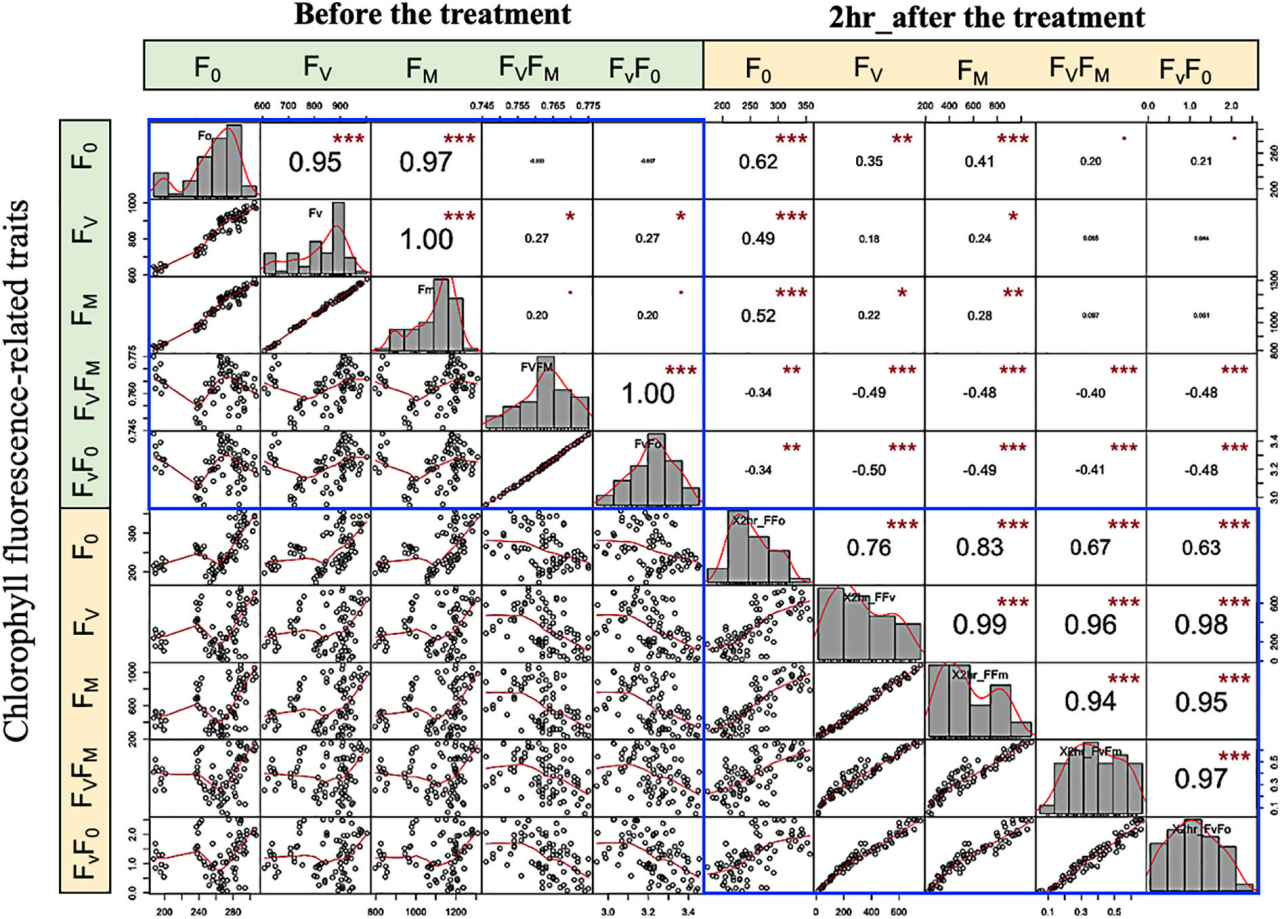
Variation and spearman scatter plots correlations among low-temperature-related traits. Histograms for F_0,_ F_V_, F_M_, F_V_/F_M,_ and F_V_/F_0_ values measured are displayed along the diagonal. The distribution of each variable is shown on the diagonal. On the bottom of the diagonal: the bivariate scatter plots with a fitted line are displayed. On the top of the diagonal: the value of the correlation plus the significance level as stars “. “, *,**, and *** are significance level P value 0.001, 0.01, 0.05, 0.1, 1 (not-significant, significant, very significant, and extremely significant, respectively).

The strength and direction of the relationship between traits can be explained by the correlation coefficient (r). The genetic correlation between chlorophyll fluorescence-related traits is assessed by means of the non-parametric spearman correlations. The correlation coefficient is presented as pairs of scatter plots with an asterisk that have a significant correlation, and the greater the correlation, the more significant. Generally, before the low-temperature stress, an extremely significant and positive correlation (P< 0.001) was observed between F_0_ with F_V_ and F_M_; F_V_ with F_M_, F_V_/F_0_, and F_V_/F_M_ (Spearman’s r > 0.95) (**Figure 6**). This finding revealed that an increase in F_0_ and F_M_ traits leads to an increase in the other variable and vice versa. Under LTS on the other hand, extreme to moderate and significant correlation between most of the studied traits. Interestingly, a positive correlation was detected between F_V_/F_0_, and F_V_/F_M,_ with all traits. These findings revealed a genetic variation and correlation between the studied phenotypic traits.

## Discussion

An understanding of genetic diversity is essential for association analysis as population structure can cause false associations (Gyawali et al., 2016). Association analysis identifies QTL by illustrating the marker-trait association that could be attributed to the strength of linkage disequilibrium between functional polymorphisms and markers within a set of diverse genotypes. This benefit is particularly great when the dataset contains traits that are time-consuming and expensive to measure. For this purpose, a cooperative trial has been formalized in Canada in which 25 to 35 lines are evaluated annually in 15 to 20 locations in Western Canada. The result is an extensive collection of historical data demonstrating a wide variety of elite barley lines that can be used to discover desirable alleles through association mapping. In this study, phenotypic correlation analysis showed that there was a significant correlation between F_V_/F_0_, and F_V_/F_M_ with all traits under LTS, suggesting relatively high genetic variability among studied genotypes (Elakhdar et al., 2018). Several DArT markers have been identified for grain quality using 91 genotypes of this panel (Beattie et al., 2010). In addition to disease ratings for true loose smut [*Ustilago nuda* (*Jens.*) *Rostr.*] and net-form net blotch [*Pyrenophora teres f. teres Drechsler*; *anamorph*: *Drechslera teres* (*Sacc*.) were recorded (Beattie et al., 2010). With such historical datasets, associations with barley yield have been identified through association mapping (Kraakman et al., 2004).

The current study is the first to investigate the genetic diversity within the Western Canadian barley cooperative trials collections using the Illumina iSelect SNP array. A set of 5063 high-quality SNP markers were identified across the barley genome (**Figure 1**). The highest number of markers were detected on the 5H chromosome (1114 SNPs), while the lowest was found on the 1H chromosome (444 SNPs). The 1H chromosome had a minimum genetic diversity level due to the low frequency of recombination rates. The findings are consistent with previous studies that used diverse markers such as SSR, AFLP, RFLP, and SNPs (Graner et al., 1991; Pakniyat et al., 1997; Backes et al., 2003; Karakousis et al., 2003; Elakhdar et al., 2016b; Amezrou et al., 2017; Bengtsson et al., 2017; Capo-Chichi et al., 2021). PIC values and genetic diversity are extremely useful for assessing the amount of polymorphism among the studied barley germplasm in breeding programs. The average PIC and GD in this study were 0.341 and 0.275, respectively (**Table 2**). The results are very similar to those of the study by Amezrou et al. (2017) using SNP markers in spring barley collection of ICARDA, while it is higher to slightly higher than that described by Bengtsson et al. (2017) and (Rodriguez et al., 2012), 0.28 in a Nordic spring barley genotypes and 0.298 in Sardinia barley landraces, respectively. Based on the type of marker, the PIC can be used to estimate the level of gene variation. In the case of multi-locus markers such as SSR, the PIC values range from 0 to 1.0. Therefore, Botstein et al. (Botstein et al., 1980), proposed three classes for the multi-allelic markers according to their PIC values; highly, moderately, and weakly informative markers in which the PIC values are > 0.5, from 0.25 to 0.5, and< 0.25, respectively. However, in the case of bi-allelic markers (such as SNPs), the maximum value of PIC is 0.5, as the marker’s scores are 50% (0) and 50% (1). Considering this aspect, the SNP markers would be classified as moderate or weak informative markers, because they are bi-allelic and also have a lower mutation rate in comparison with the SSRs (Martinez-Arias et al., 2001). This could explain the low correlation observed between genetic distances because of the low mutation rate of SNPs (Kruglyak et al., 1998). The PIC value in this study was a good indicator for studying the genetic diversity since they were moderate informative markers. Taken together, these findings provide the opportunities to identify barley genetic diversity within these geographic regions and the studied population could be used in determining alleles controlling economic traits in genome-wide association studies in the future.

### Genetic diversity within the Canadian barley collection

The genetic variance in the current studied panel indicated that our germplasm had a good genetic variation since it was collected from eight barley breeding programs resources and could be used for a different purpose. The 96 barley genotypes composed advanced breeding lines, elite germplasm and commercial varieties registered in Canada. The elite germplasms from the Western Two-Row Cooperative Registration were evaluated over 15 years. Commercial cultivars currently registered in Western Canada such as ‘AC Metcalfe’, ‘CDC Copeland’, ‘Bentley’, ‘CDC Meredith’, and ‘CDC Reserve’. Four winter barley varieties; ‘02Ab431’, ‘02Ab671’, ‘02Ab669’, and ‘2Ab08 X 05W061-208’ (Aberdeen, ID) which have been developed by the US Department of Agriculture. The lines were selected based on their high percentage of winter survival, seed quality, and seed yield. Even though the panel studied does not contain full representations of breeding materials. It reflects the majority of commonly used parental genotypes and suggests that future progress can be achieved from a limited pool of variability if new sources of genotypes are not introduced. Previously, (Elakhdar et al., 2018) reported that a mini-core collection of barley could be used for the characterization of new resource variations in breeding programs.

Bayesian clustering in STRUCTURE, PCoA and AMOVA analyses, revealed that the population might be differentiated mainly due to the different breeding program origins and by ear row type and divided into five subpopulations. Several reports show that both geographical origin and ear-row type are factors influencing barley population structure (Kolodinska Brantestam et al., 2007; Cockram et al., 2010; Bengtsson et al., 2017). Barley genotypes originating from the Coors, Cargill and AU programs, and some of the AAFRD Lacombe barley genotypes exhibited extra diverse membership. Further breeding programs displayed high stable membership in various subpopulations, although the membership attribution in each subpopulation was varied. A genetic structure that corresponds to the breeding program has previously been described in the American barley genotypes (Hamblin et al., 2010). Hamblin et al. (2010) concluded that the differentiation in breeding programs may have been influenced by breeding history and local adaptation may have contributed to this differentiation. The geographical separation of the barley genotypes observed in this study could also be explained by local adaptation resulting from alterations in day length between northern and southern regions within Western Canada. This result is very important because, in breeding programs, genetic distance is a factor that should be carefully considered in choosing the candidate parents to improve a particular agronomic trait. Despite low variation between subpopulations (5%), the partitioning value was significant (*P< 0.001*). A high level of gene flow explains the low level of diversity between subpopulations (Arora et al., 2014). Hence, preserving genetic diversity in the Western Canadian breeding programs is essential for sustained breeding advances. The latter will allow barley breeders to conserve and manage germplasm effectively in the gene bank and to use the same effect in their improvement programs.

### Patterns of linkage disequilibrium

Population geneticists have long been interested in the extent of LD since its value reveals the number of genetic markers needed to construct GWAS and the mapping resolution (Flint-Garcia et al., 2003). The LD mapping resolution impacts the extent of LD and the rate of LD decay with genetic distance across the genome (Remington et al., 2001). Genome-wide LD analyses have been previously described in different barley populations (Malysheva-Otto et al., 2006; Saisho and Purugganan, 2007; Pasam et al., 2012; Bengtsson et al., 2017). In this study, there is a clear and strong structure in the population, we, therefore, measured LD in the subpopulations assessed from the STRUCTURE analysis. The average LD decay (r^2^-value) was 0.21 (**Table 6**). The LD decayed in linkage group 1H had the lowest value of SNPs (8.79%), while the highest value (22.10%) was in linkage group 5H. With increasing genetic distance, the LD values declined to below the critical r^2 =^ 0.18 after 3.91 cM, and the same pattern was observed on each chromosome (**Figure 5**). Accordingly, the high and low levels of LD found across the whole genome provide an excellent chance to identify target QTLs in the currently studied materials (Wurschum et al., 2011). This result complies or is in agreement with an earlier study on worldwide spring barley (Pasam et al., 2012; Bengtsson et al., 2017), and European modern two-rowed barley cultivars (Kraakman et al., 2004). In our study, the 9K Barley SNP assay (5063 SNP markers) provided a region coverage of approximately 0.233 cM per SNP marker. Several association mapping analyses have been performed successfully for different traits using the 9K Barley SNP assay (Amezrou et al., 2017; Bengtsson et al., 2017; Honsdorf et al., 2017).

### The impact of breeding history on genetic diversity

Species require genetic diversity to improve to cope with environmental changes by natural selection. Genetic diversity, strength of selection, effective population size, number of generations and the mutation rate are important factors that affect the ability of populations to tolerate natural selection. Loss of genetic diversity in small populations decreases their ability to evolve to cope with environmental change, thus increasing their extinction risk. Natural selection in the short to medium term predominantly utilizes pre-existing genetic diversity, but new mutations make increasing contributions in later generations (Frankham et al., 2022). Structure analysis differentiated barley populations based on the number and frequency distributions of SNPs segregating within the populations. A simplistic model of variation in cultivated barley would suggest that the two-row type has the most diversity because it is closest to its wild ancestor which had two rows and required vernalization (Pourkheirandish and Komatsuda, 2007). On the other hand, populations of six-row and spring type have the least diversity. Both the six-row and spring-type could have been accompanied by two selective bottlenecks correlated with the creation of this gene pool (Pourkheirandish and Komatsuda, 2007). Selection and high genetic gains in barley breeding led to a loss of genetic diversity. Current genetic diversity conservation actions focus on the long-term maintenance of breeding lines under selection. Gene banks play a role in such actions by storing genetic materials for future use and the recent development of genomic information is facilitating the characterization of gene bank material for better use. The question that remains is whether the reduction in genetic diversity has affected crop production today. A case study in barley demonstrates the application of understanding relationships between genetic diversity. As an outcrossing species, barley has tremendous genetic variation. Using the barley germplasm from the Western Canadian breeding program as a case study, we inferred the potential role of re-examining the historical dataset of old genotypes for genetic diversity conservation of the current population. The complementary combination of GWAS approaches, and germplasm resources are leading to important discoveries about the relationship between genetic diversity and phenotypic variation and the impact of domestication on trait variation (Flint-Garcia, 2013).

In summary, to build on Canada’s position as a supplier of premium quality barley yield production, the Western Canadian cooperative trials breeding program aims to develop barley varieties with improved traits. Re-examine the genetic diversity within the historical genotypes is valuable when the dataset contains traits that are costly to measure and/or time-consuming, such as protein content, enzyme activity (e.g., α-amylase, diastatic power), friability, plumpness, β-glucan, and fine extract are some of the traits that influence grain quality. The recent breeding history controlled by the characteristics of the malting productions has had a more dramatic effect on the diversity of barley, entailing a decline in the diversity of the six-row spring types (Beattie et al., 2010).

## Conclusions

In the current study, genetic diversity within 96 genotypes of barley was characterized based on 5063 SNP markers. This panel represented elite barley germplasm used to study limit dextrinase and beta-glucanase. These lines have been in the Western Two-Row Cooperative Registration Tests and contain a number of barley check cultivars currently registered in Western Canada. The lines reflect the majority of commonly used parental genotypes, suggesting that further progress can be achieved from a limited pool of variability if the new resource of genotypes is not released subsequently. We have shown that the studied genotypes are genetically diverse, as well as interlinked and structured, indicating a heterogeneous collection. Although an ultimate target of breeders is the genetic diversity based on traits, our main objective was to demonstrate how SNP markers can generate biological variation between barley lines by causing differences in the recipes for proteins that are written in genes. The coverage of markers and population stratification besides the level of LD in our genotypes set was suitable to run different association mapping studies for key economic traits in barley in the future. The results obtained from this study will be useful for future genomic studies, and germplasm management in Western Canada and North American regions to elucidate genetic diversity and structure in their breeding material. Further, the results will assist the selection of parents with complementary allele combinations for future crosses and help identify progeny with the desired alleles is important to develop traits that are expensive and/or time-consuming to measure.

## Data availability statement

The datasets generated for this study can be found in FigShare, https://figshare.com/articles/dataset/Barley9Kplate3Project_96_sample_Raw_data_csv/21760586/1.

## Author contributions

LC prepared the material for genotyping. AE developed the article concept. AB prepared the individual populations corresponding to each breeding program. AE performed bioinformatics data analysis and interpretation of the results. AE wrote the manuscript. LC, TK, JN, PJ, FC, JS, GR, and AB reviewed the manuscript. All authors contributed to the article and approved the submitted version.
