# Supplementary material for: Genetic diversity and population structure assessment of Western Canadian barley cooperative trials
Source: Front Plant Sci. 2023 Jan 9;13:1006719. doi: 10.3389/fpls.2022.1006719 (PMC9868428; doi:10.3389/fpls.2022.1006719)
Supplement: Supplementary file 1 [file DataSheet_1.docx]

Supplementary Material

# Supplementary Data

**Supplemental Table 1.** Pairwise of genetic distances matrix between populations.

| **Subpopulations** | Pop1 | Pop2 | Pop3 | Pop4 | Pop5 |
| --- | --- | --- | --- | --- | --- |
| Pop1 |  |  |  |  |  |
| Pop2 | 0.279 |  |  |  |  |
| Pop3 | 0.223 | 0.693 |  |  |  |
| Pop4 | 0.279 | 0.693 | 0.693 |  |  |
| Pop5 | 0.556 | 0.122 | 0.133 | 0.133 |  |

Genetic distances calculated based on Shannon Values on 1000 permutations of pairwise populations

**Supplemental Table 2.** Estimation of number of different and effective alleles, Shannon information index and unbiased expected heterozygosity

| **Subpopulations** | **Na** | **Ne** | **I** | **uHe** |
| --- | --- | --- | --- | --- |
| 1 | 40.000 | 37.231 | 3.658 | 0.996 |
| 2 | 4.000 | 4.000 | 1.386 | 1.000 |
| 3 | 3.000 | 2.667 | 1.040 | 0.833 |
| 4 | 4.000 | 4.000 | 1.386 | 1.000 |
| 5 | 125.000 | 119.342 | 4.809 | 0.999 |

Na; No. of different alleles, Ne; No. of effective alleles, I; Shannon's information index, H; Diversity Among Pops, F; fixation index, uHe; unbiased expected heterozygosity

**Supplemental Table 3.** Percentage of variation explained by the first 3 axes in Principal Coordinate Analyses of western Canadian collections

| **Axis** | **1** | **2** | **3** |
| --- | --- | --- | --- |
| % | 11.82 | 5.76 | 4.62 |
| Cum % | 11.82 | 17.58 | 22.20 |

**
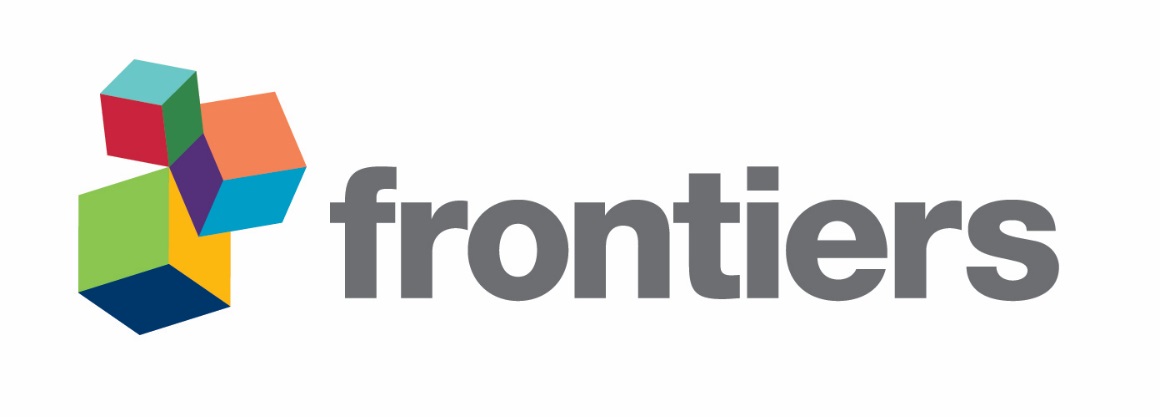
**
